# Base saturation flow rate (BSFR) and its effect on performance of pretimed signalized intersection with non-lane based urban heterogeneous traffic

**DOI:** 10.1371/journal.pone.0306112

**Published:** 2024-07-05

**Authors:** Sugiarto Sugiarto, Sofyan M. Saleh, Yusria Darma, Muhammad Rusdi, Qurrata A’yuni, Teuku Syahrul Fazila, Roudhia Rahma

**Affiliations:** 1 Civil Engineering Department, Universitas Syiah Kuala, Banda Aceh, Indonesia; 2 Center for Environmental and Natural Resources Research, Universitas Syiah Kuala, Banda Aceh, Indonesia; 3 Remote Sensing and Cartography Laboratory, Universitas Syiah Kuala, Banda Aceh, Indonesia; 4 Graduate School of Environmental Management, Universitas Syiah Kuala, Banda Aceh, Indonesia; University of Shanghai for Science and Technology, CHINA

## Abstract

Signalized intersections that are pretimed are commonly used in rapidly developing cities with diverse traffic patterns, including those found in Indonesia. These intersections are a common source of congestion and delays in road networks, particularly when there is non-lane based (NLB) traffic in urban areas. Accurate estimation of both the base saturation flow rate and capacity is essential for this type of facility, as an error in the prediction of the base saturation flow rate can result in significant bias in capacity evaluation and design at signalized intersections. The estimation of capacity at signalized intersections is critical for ensuring optimal signal timings, minimizing delay, and reducing congestion. Heterogeneous traffic, which refers to the presence of various types of vehicles with distinct static and dynamic characteristics, is a common phenomenon. To address this issue, this paper presents a modeling approach for the SFR that takes into account heterogeneous traffic and NLB movements. Indonesia, being an archipelago with 34 provinces, served as the focus of this study, which specifically concentrated on Banda Aceh, the capital province of Aceh province. Employing primary and observed data collected at a specific, predetermined signalized timing, this study aimed to investigate the impact of intersection geometry and heterogeneous traffic composition on the SFR. By adopting the modeling approach for NLB movements, the study formulated the BSFR model. To estimate the scale parameters of the BSFR, the multiple linear regression approach was utilized. The analysis results reveal that the existing BSFR based on the IHCM formula gives a substantially biased estimation because the PCEs from the Indonesian Highway Capacity Manual (IHCM) are underestimated. This source of error could be partially due to the heterogeneous (varied vehicle composition) traffic flow with NLB movements, unlike that observed under the prevailing conditions of IHCM 1997. The empirical results show that the existing IHCM should be improved to avoid overestimation, particularly for effective approach width (We) greater than 4.5 m. A comparison of the BSFR prediction model between IHCM’s PCEs and new PCEs shows that the BSFR is predicted more accurately in the latter case. This finding demonstrates that the existing IHCM can be adjusted in two ways: adjusting PCEs or calibrating the BSFR formula. The proposed models can also enhance the accuracy of BSFR prediction, leading to better signalized intersection capacity estimation, providing support for designing traffic operation, alleviating traffic congestion, and reducing congestion delay within the city.

## 1. Introduction

Pretimed signalized intersections are one of the major types of traffic facilities that significantly influence the infrastructure of road networks for efficiently operating urban traffic corridors. In terms of signalized operation, the designed capacity is vital in determining the traffic light corresponding to the cycle time and green phase at the approach of the intersection. An inefficient design capacity could lead to congestion and delay in the road network. Previous studies have been showed that congestion within a city center could lead to transport externalities, e.g., long travel times, high vehicle operating cost, air pollution, excessive energy consumption, and even serious economic loss [1–4]. Sugiarto et al. [3, 5] concluded that the facility quality of services at arterial road segments with U-turn sections and on-street parking is the major source of traffic congestion in Banda Aceh, Indonesia. Furthermore, the leading cause of traffic bottleneck is the poor trade-off between traffic demand and infrastructure supply [6]. Therefore, understanding the traffic mechanism at a signalized intersection is important for alleviating congestion. Two strategies have thus far been applied to deal with traffic breakdown at signalized intersections: improving the traffic capacity with a unique and refined design [7, 8], and decreasing the delay with optimized traffic signal control [9, 10].

In the case of non-lane based (NLB) heterogeneous traffic, the movements and compositions are generally characterized by the presence of diverse vehicle types, each with its own static and dynamic characteristics, including light vehicles, heavy vehicles, motorcycles, and rickshaws. The movements and compositions have different effects on the traffic characteristics. The Indonesian Highway Capacity Manual (IHCM, 1997) guides engineers and planners in planning, designing, and operating traffic facilities, including pretimed signalized intersections. Therefore, accurately estimating the capacity at pretimed signalized intersections plays an essential role in designing and operating such a facility.

Pretimed signalized intersections are widely operated in Indonesia, particularly in Banda Aceh, the capital province of Aceh province, Indonesia. This type of facility is a common source of congestion and delay in the road network, specifically because of the heterogeneous traffic composition and NLB movement of traffic. An insufficient formulation base saturation flow rate (BSFR) and capacity estimation for such a facility is crucial because an error in predicting the BSFR can lead to a bias in designing the capacity at signalized intersections. According to Tang et al [11], the saturation flow rate (SFR) is a vital aspect for evaluating signal timing and performance at signalized intersections. Additionally, SFR serves as a crucial input parameter for capacity calculation, signal timing design, and level of service evaluation [12, 13].

The saturation flow rate (SFR) is an important parameter in the capacity estimation, and the flow rate represents the maximum average hourly flow rate at which vehicles can pass through a stop line during green effective time [14]. Two approaches have thus far been used to estimate the BSFR: (1) Field measurement (direct method); (2) Existing formulae from traffic codes, e.g., [12] and USHCM [14, 17]. The SFR is obtained by multiplying the BSFR with adjustment factors such as the city size, side friction, grade, parking distance from the stop line, proportion of right-turning vehicles, and proportion of left-turning vehicles [15].

The BSFR estimated using the [15] formula is inaccurate because the traffic characteristics (i.e., vehicle size, vehicle type, road geometry, and signal timing) have changed considerably in recent years. Therefore, it is necessary to update the IHCM (SFR = 600W) to ensure an unbiased estimation of the capacity at pretimed signalized intersections. Improving the accuracy of capacity estimation is crucial for achieving optimal signal timings, minimizing delay, and reducing congestion. Fortunately, advanced, and recent technologies can help ensure the effectiveness of signal timing for efficient urban traffic management, such as signalized intersections that operate on conventional stage-based signal control [16]. Further advanced methods assume that vehicular movements/trajectories within the targeted intersection are simplified. The movement of vehicles at a signalized intersection was thoroughly investigated using several advanced approaches, including the extended car-following model [17]. More effort was put into modeling the trajectories of microscopic intersectional vehicular movement, which was carried out using a two-dimensional approach [18, 19]. Additionally, simulation models were used to accurately represent the path dispersion of left-turn and opposing through movement for efficient intersection design [20].

While many developing countries, such as Indonesia, still use pretimed signals and outdated traffic codes like IHCM 1997, this study proposes a comprehensive modeling technique for the BSFR in the case of pretimed signalized intersections with NLB heterogeneous traffic operation, taking into account the interactions between various factors. To develop the model, the study collected primary and observed data at three pretimed signalized intersections in Banda Aceh. The BSFR is modeled using the approach of NLB movements, and the effects of exogenous variables, such as intersection geometry and heterogeneous traffic composition, are analyzed. The multiple linear regression method with synchronous data counting proposed by [19, 21] is used to calibrate the regression parameters of the PCEs and BSFR.

The remainder of this paper is organized as follows. Section 2 reviews the existing related studies. Section 3 describes the data collection, model formulation, and estimation results obtained using a simple linear regression (SLR). Finally, Section 4 presents the discussions and conclusions of the study.

## 2. Signalized intersection capacity

### 2.1. Capacity manuals for signalized intersections

The most common capacity analysis approach for signalized intersections is the U.S Highway Capacity Manual [17, 20] traffic manual, which was recently updated in 2010 [14]. The SFR is modeled as a product of the BSFR and adjustment factors, such as non-ideal geometry, traffic, and environmental conditions, as expressed in Eq (1). The computed SFR is the “adjusted” SFR because it reflects the application of various factors that help adjust the BSFR to the specific conditions in a given intersection approach. The BSFR represents the expected average flow rate in a through-traffic lane having geometric and traffic conditions. Furthermore, the BSFR represents the saturation flow rate in a 12 ft wide traffic lane with no heavy vehicles, a flat grade, no parking, no buses that stop at the intersection, even lane utilization, and no turning vehicles. The BSFR is taken as a constant maximum flow rate at a green effective time of 1900 PCU/h/ln. The unit per lane is applied in the US because the traffic movement follows a platooning pattern, with lane-based traffic flows. This becomes a fundamental problem in the case of heterogeneous traffic with NLB movements. Thus, the traffic flow theory and manual adopted by the US cannot be directly applied to Indonesia. The local conditions must be incorporated to implement the US’s traffic code.

$$\text{S}={\text{S}}_{0}\;{\text{f}}_{\text{W}}{\text{f}}_{\text{HV}}{\text{f}}_{\text{G}}{\text{f}}_{\text{P}}{\text{f}}_{\text{BB}}{\text{f}}_{\text{A}}{\text{f}}_{\text{LU}}{\text{f}}_{\text{LT}}{\text{f}}_{\text{RT}}{\text{f}}_{\text{Lpb}}{\text{f}}_{\text{Rpb}}$$ (1)

where S represents the SFR (PCU/h/ln), S_0_ represents the BSFR (PCU/h/ln), and f terms (from left to right) represent the adjustment factors for lane width, heavy vehicles in a traffic stream, approach grade, existence of a parking lane and parking activity adjacent to lane group, blocking effect of local buses that stop within the intersection area, area type, lane utilization, presence of left-turning vehicles in a lane group, presence of right-turning vehicles in a lane group, pedestrian for left-turn groups, and pedestrian for right-turn groups, respectively.

The IHCM was proposed by the Directorate General Highways Ministry of Public Works in 1997. The IHCM is an essential tool used in the planning, design, and operation of traffic facilities in Indonesia. It was adopted from the [22] with major modifications in terms of driver behavior and fundamental road traffic characteristics. The manual was designed to allow engineers and planners to predict the quality of services of a traffic facility under a given set of traffic, geometry, and environmental conditions. According to the IHCM, the SFR is defined as the product between the BSFR (S_0_) under a set of standard conditions and the correction factors (F), which are used to consider the deviation in the actual conditions from a set of pre-determined (ideal) conditions, as shown in Eq 2.

$$\text{S}={\text{S}}_{0}\;{\text{F}}_{\text{cs}}{\text{F}}_{\text{sf}}{\text{F}}_{\text{g}}{\text{F}}_{\text{p}}{\text{F}}_{\text{rt}}{\text{F}}_{\text{lt}}$$ (2)

and the BSFR (S_0_) is defined as follows:

$${\text{S}}_{0}=600\;{\text{W}}_{\text{e}}$$ (3)

where S represents the SFR (PCU/h), S_0_ represents the BSFR (PCU/h), and f terms are the adjustment factors for the city size, side friction, grade, proportion of right-turning vehicles, and proportion of left-turning vehicles, respectively.

### 2.2. Saturation flow rate estimation approaches for heterogeneous traffic flow with NLB movements

There are continuous efforts working on the modeling and evaluating SFR and BSFR. Most of works has been done in lane-based traffic and homogenous traffic. Less of existing work has been comprehensively evaluate the BSF and its effect on capacity and level of service of pretimed signalized intersection within the heterogenous traffic, in particular in Indonesia. Tang et al [11] summarize the exiting methodology to model and evaluate SFR signalized intersection based on headway distributions. Perhaps more advance by proposing automatic estimation method for the SFR based on video detector data [23] and dynamic estimation of SFR [24]. Amongst existing research, the probability method is commonly and has been adopted by [25] to model the effects of heterogeneous traffic on the SFR at a signalized intersection by comparing the headway ratio and probability approaches. They concluded that the probability method is more appropriate to rationalize the heterogeneous traffic in India. Furthermore, Hossain [26] studied the heterogenous traffic in Bangladesh. A microscopic simulation approach was applied to model the passenger car equivalent by considering the characteristics of lane width and percentages of turning vehicles, heavy vehicles, and non-motorized vehicles.

Rahman et al. [27] showed that the capacity at a signalized intersection is substantially affected by the presence of non-motorized traffic and rickshaws in the city of Dhaka, Bangladesh. They concluded that the capacity decreases in the presence of non-motorized traffic and rickshaws. They recommended adjusting the passenger car equivalent for non-motorized traffic and rickshaws to better predict the signalized capacity. Chu and Sano [28] examined the traffic conditions in two emerging cities, namely Hanoi and Bangkok. They proved that the SFR is strongly influenced by the percentage of two-wheelers: the higher the percentage of two-wheelers, the lower the observed capacity. The regression approach has been used to model the passenger car equivalent of two-wheelers and BSFR.

Patil et al. [29] conducted a study in the city of Mumbai, India. The authors modeled the SFR using linear regression with exogenous variables such as the effective width and percentages of heavy vehicles and turning vehicles. Zhang and Chen [30] calibrated the USHCM to model the traffic in the city of Nanjing, China. They showed that after calibrating with the characteristics of local traffic conditions, the USHCM (2000) could be used as a guide to design and operate signalized intersections in China. Nguyen [31] introduced the term motorcycle homogenous traffic to analyze traffic flows dominated by two-wheelers at signalized intersections in Hanoi, Vietnam. The motorcycle unit (MCU) is used as the unit to measure the SFR at a signalized intersection. They implemented a linear regression approach to model the BSFR at a signalized intersection and found that the value of the two-wheeler unit for a car is 3.67 MCU.

Anusha et al [29] calibrated the HCM (2000) using observed data from three signalized intersections in the city of Bangalore, India. They revealed that the high percentage of two-wheelers produced a large deviation in the SFR between the observed and estimated values when using the HCM (2000). Furthermore, a robust association is evident between the quantified saturation flow and the proportion of two-wheeler traffic, which indicates that two-wheelers exert a substantial influence and should be considered in the assessment of signalized intersection capacity [32]. The two-wheeler and heavy vehicle adjustment factors were introduced and tested. They concluded that the HCM (2000) can be used after considering these adjustment factors. Perhaps the most advanced approaches applied for modeling signal timing intersections considering environmental factors include integrated optimization of traffic quality, emissions, and fuel consumption [33]; application of car-following model to the analysis of vehicle exhaust emissions [13]; and PCU and SFR for highly heterogeneous traffic at urban signalized intersections [34].

Previous studies have shown that adopting the HCM traffic manual requires a calibration using local adjustment factors. Several researchers from developing countries concluded that heterogeneous traffic flow and complex traffic movements (NLB) present complex situations and that they cannot be explained using the traffic flow theory adopted by a developed country. Consequently, conducting studies to model theories using locally observed data is a prerequisite. In this context, this research is an initial contribution providing a basic empirical traffic theory and modeling approach for heterogeneous traffic with NLB movements, particularly at signalized intersections with pre-time signal timings. The empirical models established in this study can help conclude whether the existing IHCM (1997) [15] is relevant to current conditions or whether it requires upgradation/calibration.

## 3. Methods

### 3.1. Location of study and data collection

The study was conducted in city center of Banda Aceh, a capital city of Aceh Province, the most western part of Indonesia. The target locations from where the observed traffic data were collected, including three pretimed signalized intersections located in Banda Aceh city with various geometrical characteristics and vehicle compositions. Four-legged pre-timed signalized intersections were considered as the target traffic facilities:

- MAN Jambo Tape intersection (5°33’46"N; 95°19’47"E);
- PUPR intersection (5°32’03"N; 95°18’14"E);
- BPKP intersection (5°33’11"N; 95°20’40".

**Fig 1:**
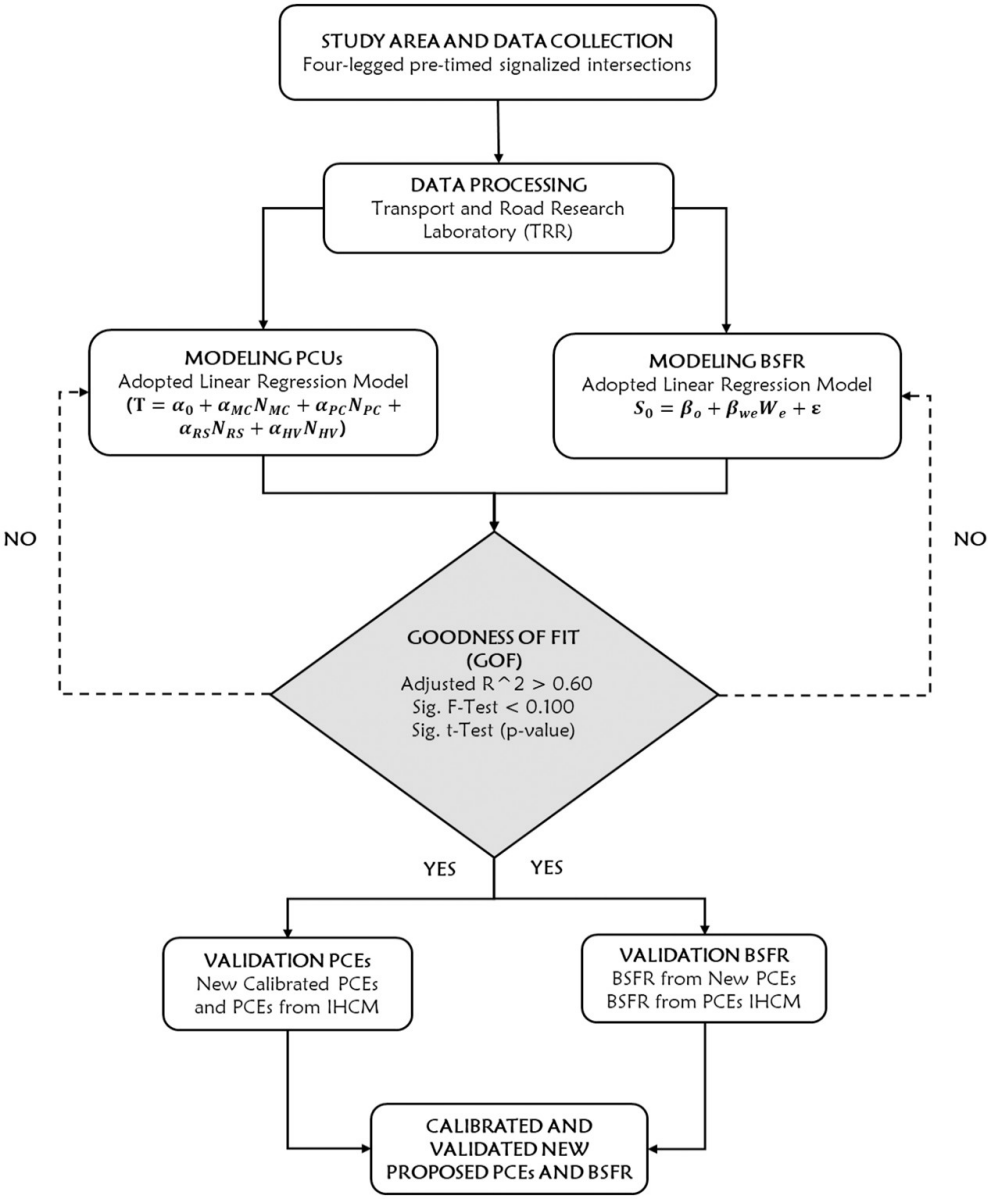
Framework diagram of the analysis.

These intersections were selected because they have moderate traffic volumes and approach widths in the range of 2.8–8 m. Additionally, this research study was carried out through a series of stages that are visually illustrated in Fig 1, which displays the research diagram.

Regarding data collection, first, the geometric conditions, such as the effective approach width, lane width, stop line position, dimensions of the intersection; including conflict area; and presence of left-turn on red (LTOR), were measured directly on site. Second, the traffic stream data, namely the numbers of straight movements, right turns, left turns (if not permitted LTOR), and the type of vehicle for each movement, were observed directly on site during the morning peak (07:15–08:50) and evening peak (16:30–18:00). Lastly, the signal timing data, such as green interval, red interval, yellow interval, star-lag time, and end-lag time, were measured directly on site corresponding to the morning and evening peak hour observations.

The geometric data were obtained by direct measurement at the target intersections by surveyors. The observation was conducted during off-peak hour traffic to avoid difficulties due to vehicle movements. The geometric data were recorded by filling survey forms including drawing the layout of the intersections. The video camera was utilized to capture the traffic data for each approach. The cameras were placed on a tripod or mounted on a high-rise building at the survey location. During morning and evening observations, 40 cycles of data were extracted for each approach. As a result, a total of 480 cycles of data were used in this study. Additionally, the signal timing data, including the start-lag and end-lag times, were directly measured on-site using a stopwatch and recorded on the survey form. The start-lag or lost time was recorded from when the green phase appeared until the driver in the front queue reacted, assuming that 2–3 cars or 1–2 two-wheelers have been discharged from the stop line. Conversely, the end-lag time was recorded from when the yellow light was displayed until all vehicles in the queue were discharged from the stop line.

### 3.2. Data processing

Three common methods have been used to model the BSFR: (1) Transport and Road Research Laboratory [35, 36]; (2) The Headway Ratio [14, 22]; (3) Regression model [21, 36–38]. The Headway ratio method is the easiest and most widely applied method by engineers. However, for heterogenous traffic flow with NLB movements, this method would lead to substantial bias. The NLB movements in heterogeneous traffic are complex and irregular and characterized by limited gaps and bumper-to-bumper movement, making it difficult to observe the headway between the following vehicles. In such cases, where traffic is arbitrary and does not follow a lane discipline system, the regression approach is simpler.

In this study, a multiple linear regression approach [21, 37, 39], namely the synchronous regression (SR) formerly proposed by [21, 37], is implemented. According to Branston and Zuylen [21]. The modeling consists of (1) Calibration of the regression parameters PCU and BSFR; (2) Simulation of the estimated scale parameters (posterior) using Monto Carlo simulation by accommodating 1,000 iterations to ensure the efficiency and consistency of the regression parameters in the posterior model.

### 3.3. Modeling PCUs

The SR approach is utilized to model and determine PCUs in this study. The PCU is a unit used to convert the various types of vehicles (heterogeneous traffic) into homogenous ones in terms of passenger car equivalent (PCE). To this end, the saturation time during green displays is regressed to total traffic compositions observed during green effective (saturated time). To ensure saturated flow, the terms of the saturation flow must be clearly defined first. The saturation time is the length of the period observed during maximum discharge flow that could be maintained during green effective onset. According to on-site observation, the initial lost time (start-lag) is in the range of 4–5 s while the end-lag time (additional time after yellow onset) is approximately 3 s. It should be noted that during end-lag, the field observation showed that the discharge flows are continuing until the red phase appears. Therefore, the saturation time in this study is presumed to be the sum of the effective green time and end-lag time. The observed vehicle composition represents heterogeneous traffic comprising motorcycles (MC), passenger cars (PC), rickshaws (RS), buses (B), and medium trucks/buses (TR/BS). Accordingly, the regression formula can be expressed as follows:

T=∑αN+ε (4)

$$\text{T}=\alpha_{0}+\alpha_{\text{MC}}{\text{N}}_{\text{MC}}+\alpha_{\text{PC}}{\text{N}}_{\text{PC}}+\alpha_{\text{RS}}{\text{N}}_{\text{RS}}+\alpha_{\text{HV}}{\text{N}}_{\text{HV}}$$ (5)

The PCE can be calculated as:

$${\text{PCE}}_{\text{i}}=\frac{\alpha_{\text{i}}}{\alpha_{\text{PC}}}$$ (6)

where T is the observed saturated time (s), N represents the observed number of vehicles discharged during the saturated time for MC, PC, RS, HV, α_0_ is start lost time, α represents headway corresponding to the type of vehicles of motorcycle (MC), passenger car (PC), rickshaw (RS), bus (BS), and heavy vehicle (HV: bus and truck), α_PC_ is headway corresponding to passenger car (PC) and ε represents a systematic disturbance of the regression model, which is assumed to be normally distributed.

### 3.4. Modeling the base saturation flow rate (BSFR)

As the primary objective of this research is to fine-tune the existing Single Frequency Receiver (SFR) model within the IHCM 1997, a simple linear model (similar to the one employed in IHCM) is implemented to adjust the SFR and PCEs. In this case, the BSFR was formulated from the SR approach proposed by Branson and Zuylen (1978) and Branson and Gipps (1981). Two regression models were developed: (1) simple model and (2) multiple models. The simple model is considered because the calibrated model will be used to validate the existing IHCM BSFR model (S_0_ = 600W_e_) formulated using the simple model. Furthermore, the multiple models are applied when additional exogenous variables are added, e.g., the percentages of vehicle compositions and vehicle movements. The basic form of the BSFR model is as follows:

$${\text{S}}_{0}=\beta_{\text{o}}+\beta_{\text{we}}{\text{W}}_{\text{e}}+\varepsilon$$ (7)

$${\text{S}}_{0}=\beta_{\text{o}}+\beta_{\text{we}}{\text{W}}_{\text{e}}+\sum \beta_{\text{i}}{\text{f}}_{\text{i}}+\varepsilon$$ (8)

where S_0_ is the observed BSFR (PCU/h), W_e_ represents the effective width, and β_i_ is the scale parameter corresponding to the type of exogenous variable f_i_ (i.e., lane width, percentage of vehicle compositions, and percentage of vehicle movements).

## 4. Results and discussions

### 4.1. Traffic compositions and calibration for PCUs

**Fig 2:**
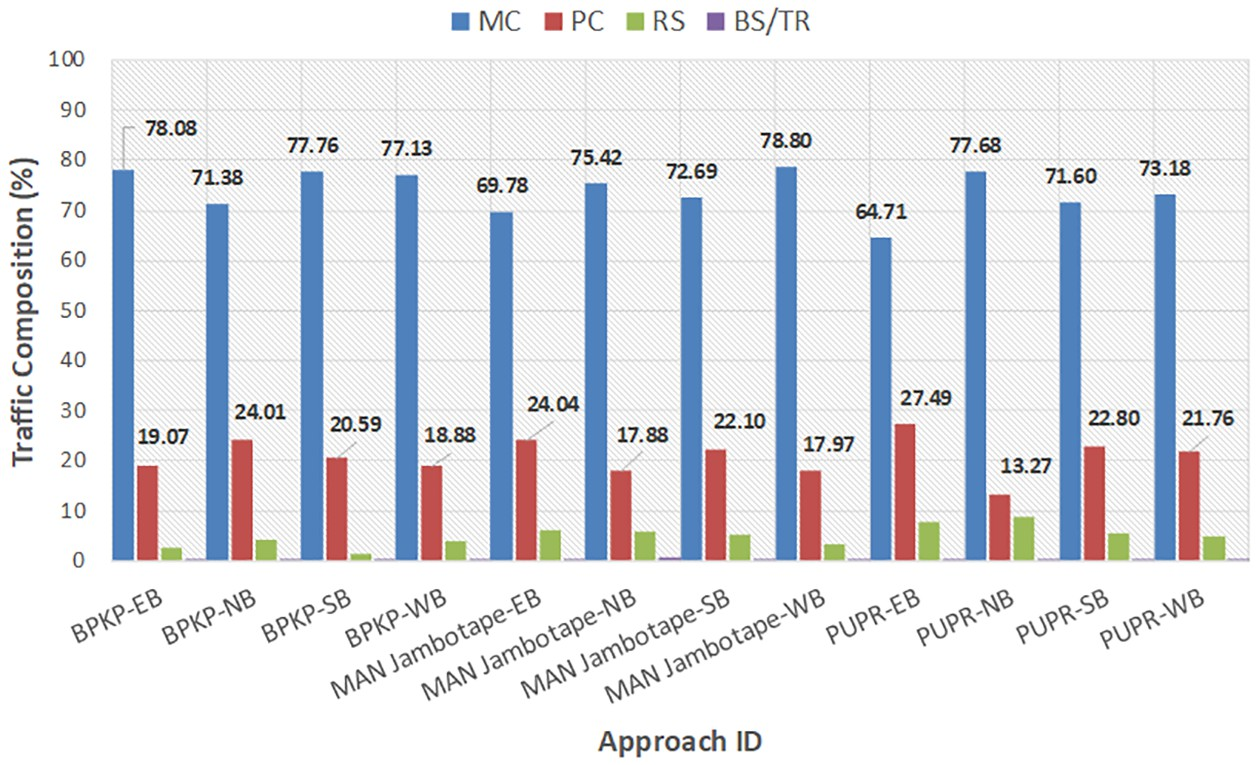
Extracted vehicle composition at target signalized intersections.

Fig 2 shows the traffic composition at the target signalized intersections. The tendencies of the traffic compositions seem considerably the same for all the approaches. The most dominant type of traffic was motorcycles (MC), accounting for approximately 74% of the traffic on average, followed by passenger cars (PC) accounting for up to 21% on average. The remaining traffic was rickshaws (RS) and buses/trucks (HV), accounting for 5% of the traffic. It should be noted during peak hour traffic the heavy vehicles (bus and truck) are restricted from entering the city center, therefore the HV in this study is excluded from the analysis.

Table 1 lists the calibrated PCUs using the saturation time as the dependent variable regressed to an exogenous variable of the observed vehicle composition during green effective. A total of 360 cycles of dataset are used to predict the average headway of each type of vehicle (MC, MC and RS). Moreover, the PCEs of each type of vehicle can be calculated using the ratio of the headway of each vehicle to the PC headway using Eq (6). The result of the PCEs calculated according to the average headway are listed in Table 1. Table 1 indicates that the adjusted R^2^ and significant F-test for ANOVA show a moderate goodness of fit (0.6 < R^2^ < 0.8; Sig. F-test at 0.00% level of error). The scale parameters are assigned with statistically significant estimated coefficients as they show greater p-value (≤ at 5% error level). In Table 1, N/A represents the variable whose statistics are grossly insignificant at a 10% level of error. This insignificant variable belongs to heavy trucks and buses, specifically at the location of PUPR and MAN Jambo Tape. This is partially because the volumes of heavy trucks and buses are considerably low during peak hour observation at these locations.

**Table 1:** Calibrated headway parameters using regression.

Parameter	Calibrated Parameters				Adjusted R^2^	Sig. F-Test	Cycles of Sample
	α_0_	α_MC_	α_PC_	α_RS_			
Coefficient	9.500	0.136	0.562	0.440	0.69	0.000	480
t value	32.50	10.66	13.40	4.73			
sig. P-value	0.000	0.000	0.000	0.000			

The PCEs, listed in Table 2, are acceptable for MC and RS as statistical significance of the predicted headway for MC and RS are acceptable. The validation of the predicted PCEs (new PCEs) in this study was performed by comparing the new PCEs with the PCEs from IHCM.

**Table 2:** Estimated PCEs for observed vehicles.

Vehicle Type	PCEs Values
Motorcycle (MC)	0.24
Passenger Car (PC)	1.00
Autos Rickshaw (RS)	0.78

**Fig 3:**
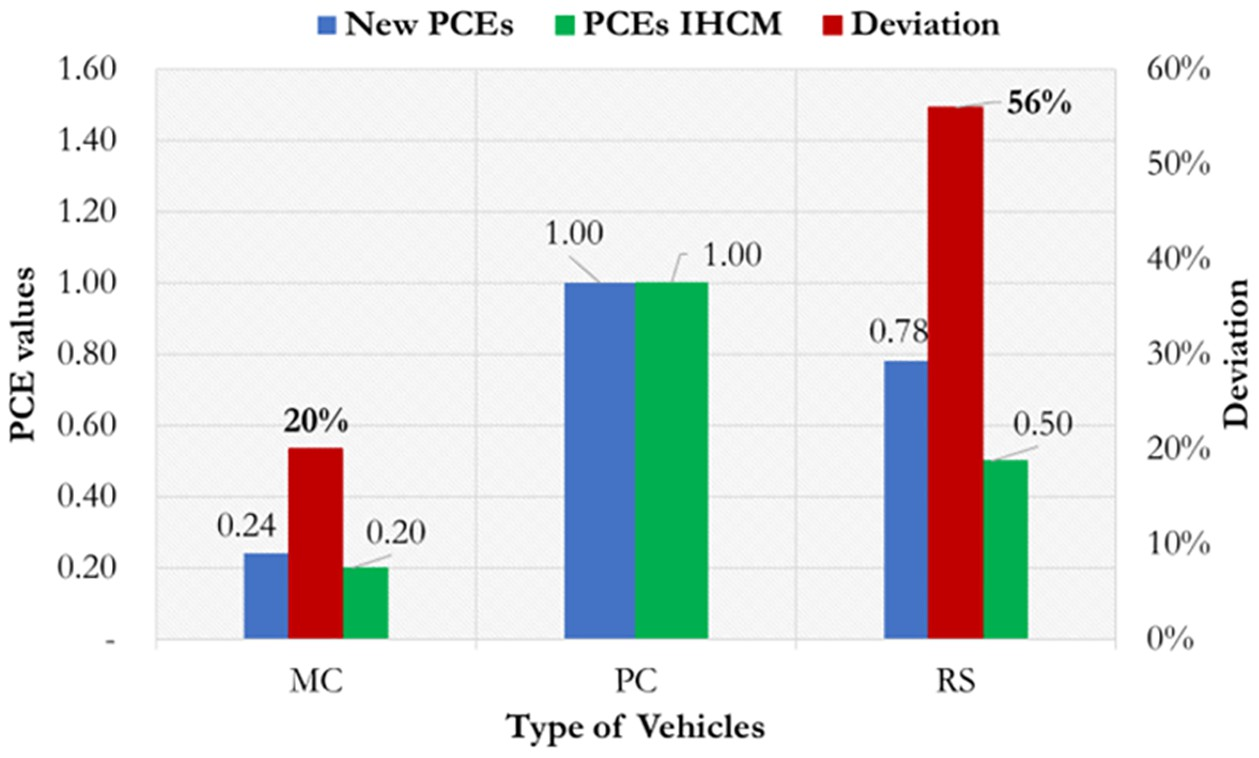
Validation results between new PCEs and PCEs from IHCM.

Fig 3 shows the validation result between the new PCEs with PCEs obtained from the IHCM. The validation results reveal that a larger deviation is found for PCE of rickshaws (RS) accounting up to 56%. It probably due to the current operated RS is motorized RS or so-called autos RS and the dimension of current RS also significantly larger compared to conventional RS. Furthermore, the deviation among new PCEs and IHCM PCE is due to the differences in the physical size (vehicle dimension), intersection geometry conditions, and vehicle compositions at present compared to the year 1997 when the IHCM was published by the government. Moreover, drivers’ performance is expected to be different compared to the condition when the IHCM was investigated. These significant changes in the PCEs could substantially affect the prediction of the BSFR for signalized intersections. Moreover, as the traffic circumstances are largely overflowing with more than 70% of two-wheelers, the PCEs for MC would drastically alter the BSFR value obtained using the IHCM formula. Thus, it can be concluded that the PCE value provided by IHCM should be adjusted with the present prevailing conditions.

### 4.2. Calibrated model for the BSFRs

Tables 3 and 4 list two calibrated BSFR models for BSFR using PCEs from IHCM and BSFR using new PCEs, respectively. Two simple models were formulated and calibrated in this study: the first model is without intercept and the second is with intercept. Practitioners can more easily apply the simple model for validation. As for the first model without intercept, the dependent variable is only regressed to the exogenous variable of effective width (W_e_). The second model accommodates the intercept and effective width as regressors. For calibrating the BSFR model, 360 cycles of observed data comprising eight intersection approaches/legs were used. It should be noted that the observed BSFR data for the calibration process are distinguished into BSFR using the PCEs from IHCM (1997) and the BSFR obtained using the new PCEs calibrated in this study, as listed in Table 2. The purpose of this distinction is to validate the existing BSFR formula by IHCM and check whether it can still be used for the design and operation of signalized intersections in Banda Aceh.

**Table 3:** Calibrated BSFR model using PCEs from IHCM.

Model Formulation	Parameter	Calibrated Parameters		Adjusted R^2^	Sig. F-Test	Cycles/Approaches
		β_0_	β_1_			
Model 1 (S_o_ = 493W_e_)	Coefficient	N/A	493	0.99	0.000	480/12
	Sig. P-value	N/A	0.000			
Model 2 (S_o_ = 783 + 368W_e_)	Coefficient	783	368	0.97	0.000	480/12
	Sig. P-value	0.000	0.000			

**Table 4:** Calibrated BSFR model using new PCEs.

Model Formulation	Parameter	Calibrated Parameters		Adjusted R^2^	Sig. F-Test	Cycles/Approaches
		β_0_	β_1_			
Model 1 (S_o_ = 622W_e_)	Coefficient	N/A	622	0.99	0.000	480/12
	Sig. P-value	N/A	0.000			
Model 2 (S_o_ = 1,020 + 459W_e_)	Coefficient	1,020	459	0.93	0.000	480/12
	Sig. P-value	0.000	0.000			

Tables 3 and 4 implies that the adjusted R^2^ and significant F-test for ANOVA show excellent goodness of fit (R^2^ > 0.9; Sig. F-test at 0.00% level of error). The scale parameters are assigned with statistically significant estimated coefficients as they show greater p-values, accepted at a 1% error level. In Tables 3 and 4, N/A represents the model with intercepts excluded from the model (regressed without intercept).

### 4.3. BSFR based on PCEs from the IHCM

**Fig 4:**
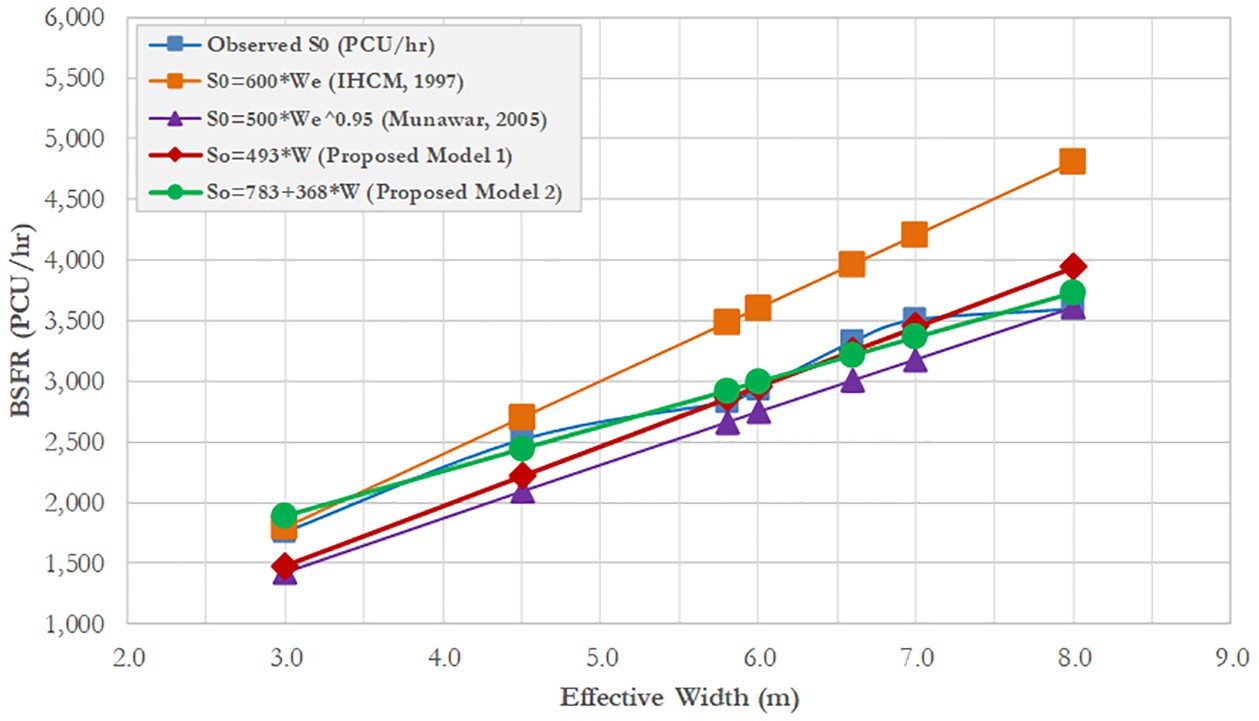
Comparison of proposed BSFR model based IHCM’ PCEs with that predicted based on IHCM.

The two proposed BSFR models based on the PCEs from IHCM were validated by plotting and comparing between the proposed models, IHCM model, and Munawar’s Model (2005). The purpose of this validation is to test whether the existing IHCM model can predict the BSFR or would produce a biased estimation, by comparing it with the observed BSFR. To ensure the prediction efficiency of the IHCM model, the previous model developed by Munawar (2005) is used to compare with the proposed models. Fig 4 shows the validation result proposed by the BSFR and IHCM models based on PCEs from IHCM.

Fig 4 shows that the BSFR-based IHCM’s formula (S_0_ = 600W_e_) and IHCM’s PCEs lead to overestimation compared with the observed BSFR, proposed models, and Munawar’s model. Although the BSFR from the IHCM was overestimated, the existing model gives a valid estimation for approach widths in the range of 3–4.5 m. The proposed model 1 (S_0_ = 493W_e_) gives a valid estimation only for effective widths (W_e_) in the range of 6–8 m. The proposed model 2 (S_0_ = 783 + 368W_e_) gives a good estimation, as the prediction plotting line is close to the observed data. Munawar’s model has the lowest prediction accuracy for all effective widths. This could be due to the exponential form of the BSFR, leading to a poor fitting of the dataset. In conclusion, the IHCM should be corrected to avoid the overestimation resulting from its BSFR formula, particularly for effective approach widths greater than 4.5 m.

To measure the validity, the BSFR model results based on the IHCM’ PCEs are plotted between the predicted and observed values corresponding to the IHCM model, proposed models, and Munawar’s model. The root-mean-square error (RMSE) and root-mean-square percentage error (RMSPE) were employed. The RMSE measures the degree of disturbance between two datasets. In other words, it compares the predicted and observed values. The lower the RMSE value, the closer the predicted and observed values; and the corresponding model gives the best fit. Furthermore, the tangent of a 45° line is utilized to measure the similarity between the predicted BSFR on the vertical axis and the observed BSFR on the horizontal axis.

**Fig 5:**
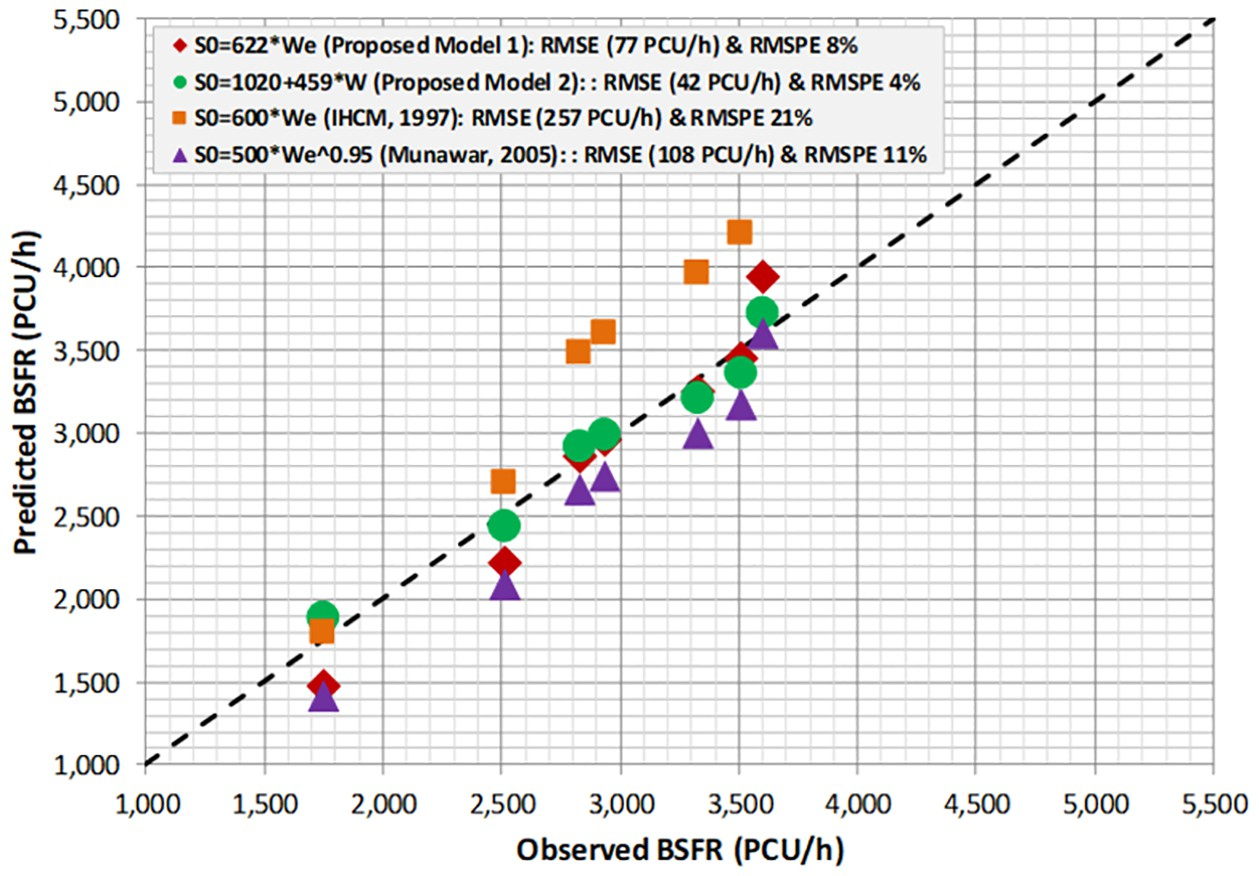
Validation proposed BSFR model based IHCM’ PCEs.

Fig 5 represents the visualization of a 45° line between the predicted and observed BSFR across four models. The 45° line-plot demonstrates that the predicted and observed datasets are randomly distributed around the 45° line. The closer the predicted and observed data, the better the BSFR model. Furthermore, the proposed model 1 (S_0_ = 493W_e_) and model 2 (S_0_ = 783 + 368W_e_) are the closest models compared to IHCM (S_0_ = 600W_e_) and Munawar (S_0_ = 500 W_e_^0.95^). To prove the statistical disturbance between the predicted and observed BSFR, the measured RMSEs indicate that the IHCM model (S_0_ = 600W_e_) has the highest RMSE of approximately 257 PCU/h (RMSPE 21%) compared with the proposed model 1 (S0 = 493We) and proposed model 2 (S_0_ = 783 + 368W_e_), accounting for 77 PCU/h (RMSPE 8%) and 42 PCU/h (RMSPE 4%), respectively. To sum up, the existing BSFR based on the IHCM formula exhibits a substantially biased estimation.

### 4.4. BSFR based on new proposed PCEs

**Fig 6:**
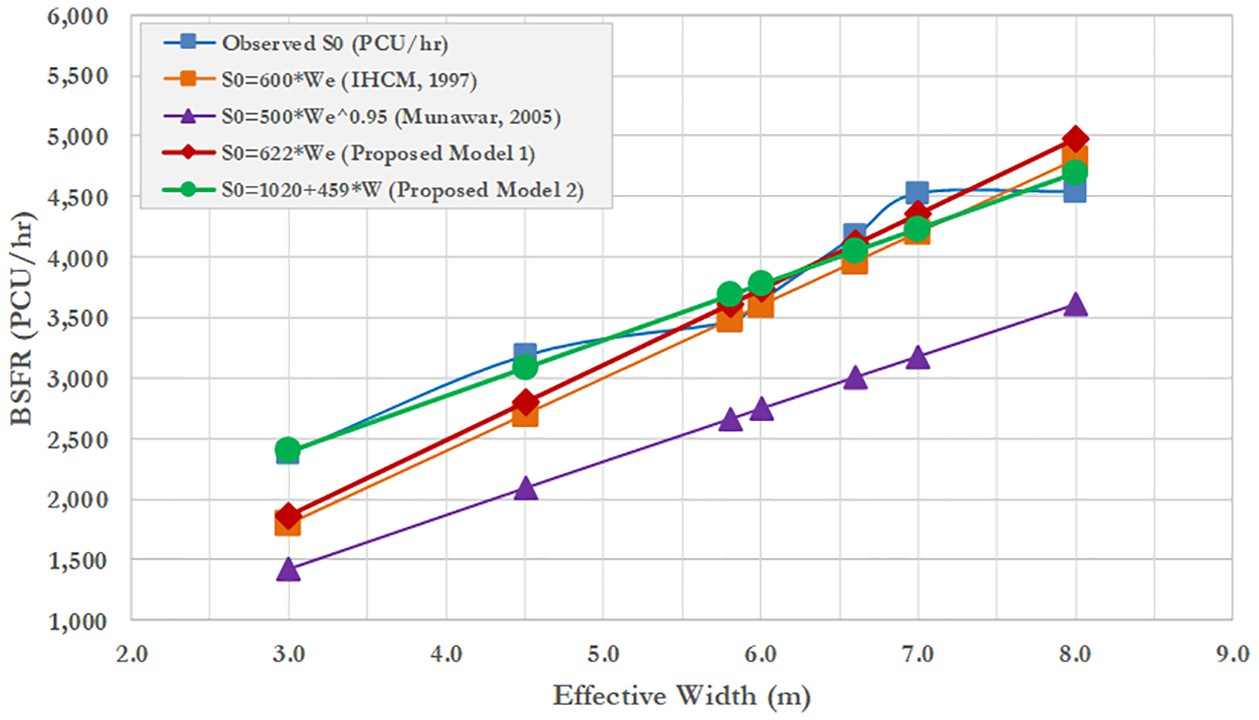
Comparison between predicted and observed BSFR values across BSFR models based on new PCEs.

Fig 6 shows that the BSFR based on IHCM’s formula (S_0_ = 600W_e_) obtained by considering new PCEs is superimposed on the results obtained using the proposed model 1 (S_0_ = 622W_e_). The predicted models for both IHCM and proposed model 1 are well-fitted, particularly for effective widths in the range of 5 to 8 m as the observed BSFR approaches the predicted value from the IHCM and proposed model 1. The proposed model 2 (S_0_ = 1020 + 459W_e_) seems appropriate for narrow effective widths (3–5 m). Overall, the adjustment of new PCEs could improve the prediction of BSFR obtained from the IHCM formula.

**Fig 7:**
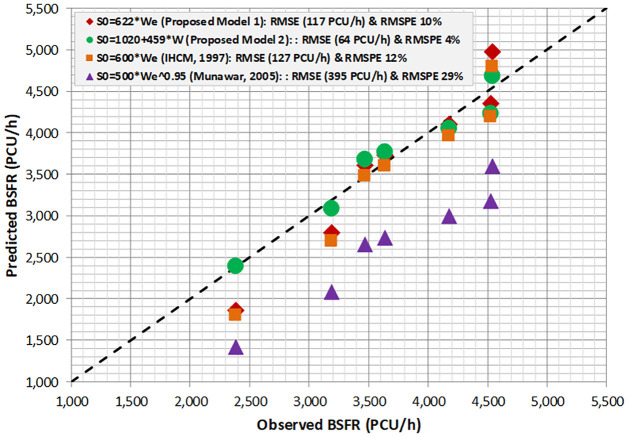
Validation proposed BSFR model based new PCEs.

Fig 7 characterizes the visualization of a 45° line between the predicted and observed BSFR across four models considering the new PCEs. The 45° line-marking proves that the predicted and observed BSFR data are randomly scattered over a 45° plotting line. Moreover, this verifies that the proposed model 1 (S_0_ = 622W_e_) and model 2 (S_0_ = 1020 + 459W_e_) are the closest models compared to IHCM (S_0_ = 600W_e_) or Munawar’s model (S_0_ = 500 W_e_^0.95^). The statistical error between the predicted and observed BSFR indicates that the IHCM model (S_0_ = 600W_e_) has the highest RMSE of approximately 127 PCU/h (RMSPE 12%), followed by proposed model 1 (S0 = 622We) and proposed model 2 (S_0_ = 1020 + 459W_e_), which show RMSE values of 117 PCU/h (RMSPE 10%) and 64 PCU/h (RMSPE 4%), respectively.

Section 4.3 and 4.4 of the study focused on comparing the efficiencies of BSFR used in the current IHCM and the newly proposed BSFR and PCEs. The aim was to show their impact on the prediction of BSFR in the unit of PCU/h. The base saturation flow rate (BSFR) was also taken into account to demonstrate the effect of PCEs on the performance of pre-timed signal intersection. However, the BSFR-based IHCM’s formula (S0 = 600We) led to overestimation compared to the observed BSFR and proposed new BSFR. The proposed model (S0 = 493We) provided a valid estimation, particularly for effective widths (We) ranging from 6 to 8 meters. This more accurate BSFR could enhance the capacity of signalized intersection and improve the efficiency of signal timing operation.

## 5. Summary and conclusion

The main objective of this study was to investigate the potential of a regression technique to establish and determine new PCEs and a formula for the base saturation flow rate (BSFR) at pre-timed signalized intersections, considering the prevalence of heterogeneous traffic and non-lane-based movements in emerging countries like Indonesia. This paper introduces a straightforward model for predicting the PCEs and BSFR at a signalized intersection based on vehicle compositions and approach/effective widths. The regression method achieved satisfactory results for the PCE model and exceptional outcomes for the BSFR models. The vehicle composition significantly impacted the determination of PCEs, while the effective width played a critical role in predicting the BSFR. The proposed BSFR models can serve as a valuable tool for minimizing prediction bias and highlighting the primary source of bias in the BSFR formula provided in the IHCM. The key findings and contributions of this study are as follows:

- A significant deviation is found between new PCEs and the PCEs obtained from IHCM. A greater deviation is found in the PCE rickshaws (RS) accounting for up to 56%. This deviation is largely due to the differences in the physical size (vehicle dimension), drivers’ performance, and vehicle compositions at present. This finding shows that the PCEs used in IHCM require adjustment with present prevailing traffic conditions.
- Considering the PCEs from IHCM, the BSFR values obtained based on the IHCM’s formula (S0 = 600We) and IHCM’s PCEs were overestimated compared with the BSFR obtained using the proposed models. The proposed model 1 (S0 = 493We) gave a valid estimation for effective widths (We) in the range of 6–8 m while the proposed model 2 (S0 = 783 + 368We) gave a valid estimation at narrow widths. The existing IHCM should be improved to avoid overestimation, particularly for effective approach widths greater than 4.5 m. The result of the statistical disturbance between the predicted and observed BSFR, such as RMSE and RMSPE, shows that the existing BSFR based on the IHCM formula gives a substantially biased estimation (RMSE 257 PCU/h, RMSPE 21%).
- Considering new PCEs, the BSFR based on the proposed model 1 (S0 = 622We) and IHCM (S0 = 600We) gave a valid prediction for effective widths in the range of 5–8 m while the proposed model 2 (S0 = 1020 + 459We) seems to be valid for narrow effective widths (3–5 m). The adjustment of the new PCEs helped correct the prediction of BSFR obtained from the IHCM formula. The statistical error between the predicted and observed BSFRs indicates that the IHCM exhibits a better RMSE of approximately 127 PCU/h (RMSPE 12%), followed by proposed model 1 (S0 = 622We) and proposed model 2 (S0 = 1020 + 459We), which show RMSE values of 117 PCU/h (RMSPE 10%) and 64 PCU/h (RMSPE 4%), respectively.

In summary, the existing BSFR based on the IHCM formula produces a substantially biased estimation considering the PCEs in IHCM. This source of error could be partially due to heterogeneous (varies vehicle composition) traffic flow with NLB movements at present. Thus, adjustment to the BSFR formula used in the IHCM is essential. Furthermore, a comparison between the BSFR values obtained using the PCEs from IHCM and new PCEs showed that the BSFR can be better predicted in the latter case. Therefore, the IHCM code can be corrected in two ways: adjusting the PCEs in the IHCM or calibrating the scale parameters in the BSFR formula given in the IHCM. The main objective of this preliminary study is to acknowledge a limitation in the data collection process. Specifically, the data collected was only gathered from three of the busiest intersections in Banda Aceh, which is the westernmost province in Indonesia. The current investigation seeks to demonstrate that the current traffic conditions are substantially different from those in 1997, as suggested by IHCM. To enhance the comprehensiveness and systematic nature of the study, it is recommended that data be collected from other cities in Indonesia to create a more robust dataset. Moreover, advanced modeling techniques, such as Bayesian and machine learning approaches, can be employed to surmount the limitation of a small data set and achieve more accurate empirical results.
